# Surveillance and Control of Antibiotic Resistance in the Mediterranean Region

**DOI:** 10.4084/MJHID.2016.036

**Published:** 2016-07-01

**Authors:** Walter Ricciardi, Gabriele Giubbini, Patrizia Laurenti

**Affiliations:** Institute of Public Health - Section of Hygiene, Università Cattolica del Sacro Cuore, Largo Francesco Vito, 1-00168, Rome, Italy

## Abstract

Antibiotic resistance is one of the most relevant problems in the healthcare: the growth of resistant microorganisms in healthcare settings is a worrisome threat, raising length to stay (LOS), morbidity and mortality in those patients.

The importance of the antibiotic resistance and its spread around the world, gave rise to the activation of several surveillance systems, based especially on the collection of laboratory data to local or national level.

The objective of this work is to carry out a review of the scientific literature existing on the topic and scientific activities related to surveillance of antibiotic resistance in the countries bordering the Mediterranean Sea.

Recent Data from European Centre for Disease Prevention and Control (November 2015) show, for different combinations bacterium-drug, an increase of resistance from North to South and from West to East of Europe. It is of particular concern the phenomenon of resistance carried out by some gram-negative, specifically *Klebsiella pneumoniae* and *Escherichia coli* to third-generation cephalosporin, often combined in opposition to fluoroquinolones and aminoglycosides.

Is particularly high the incidence of resistance to carbapenems by strains of *Enterobacteriaceae* (*Klebsiella* included). The resistance exerted by MRSA (Methicillin-resistant *Staphylococcus aureus*) continues to be relevant, albeit showing some decline in recent years. The incidence of resistance carried on by *Streptococcus pneumoniae* is stable and is mainly relevant to macrolides. Finally, a significant increase in recording relatively exercised by *Enterococcus faecium* to Vancomycin.

Detecting, preventing, and controlling antibiotic resistance requires strategic, coordinated, and sustained efforts. It also depends on the engagement of governments, academia, industry, healthcare providers, the general public, and the agricultural community, as well as international partners. Committing to combating antibiotic-resistant microbes does support patient care, economic growth, agriculture, and economic and national security.

## Introduction

Antibiotics are medicaments used for treating infections due to bacteria.

Unfortunately, when bacteria are exposed to antibiotics can develop mechanisms to survive the action of those drugs. In this situation, antibiotics may be partially or utterly ineffective and illnesses could become arduously treatable and, in some cases, untreatable.

The inappropriate and excessive use of this drugs is the leading cause of the enlargement of the phenomenon. Antibiotic resistance is one of the most relevant problems in the healthcare: the growth of resistant microorganisms in healthcare settings is a worrisome threat, raising length to stay (LOS), morbidity and mortality in those patients.

Antibiotic misuse includes: taking antibiotics when not needed; taking antibiotics through ways other than prescription; skipping doses; self-medicating; antibiotic sharing; irregularities in the intervals of taking the drug.

Inappropriate prescribing includes: unnecessary prescription; improper prescription of broad-spectrum drugs; prescription of an unsuitable antibiotic; inadequate dosage and/or duration of therapy.

Therefore, prevention of resistance is a key weapon. It is important to target therapeutic interventions, to review and to implement surveillance.

The importance of the antibiotic resistance and its spread around the world, gave rise to the activation of several surveillance systems, based especially on the collection of laboratory data to local or national level.

The objective of this work is to carry out a review of the scientific literature existing on the topic and scientific activities related to surveillance of antibiotic resistance in countries bordering the Mediterranean Sea. The Mediterranean basin includes some twenty countries with substantial differences regarding size, population, and culture. In addition, these countries have very different socio-economic conditions with marked differences between the European countries of Northern Mediterranean and African and Asian countries of South Mediterranean. Of course, health care is affected by these socio-economic situations and the European countries of the Mediterranean have a higher level health care. Despite this, the active exchange of people and goods between the countries of the Mediterranean has always influenced some interdependence concerning both infections and antibiotic resistance of bacteria.

We tried to make an update of the existing data on the subject matter, using the most reliable sources (PUBMED).

## Methods

This work results from a thorough study of existing literature on surveillance data on antibiotic resistance in countries bordering the Mediterranean Sea.

In particular, it compares the situation described by the most recent data available, with the situation instead drawn from data dating back to about a dozen years does.

Until 2010, the working group leading the “ARMed (Antibiotic Resistance Surveillance and Control in the Mediterranean Region) project” (who started in 2003, financed by the European Commission’s Directorate General for Research) had conducted relevant and interesting scientific studies on surveillance of antibiotic resistance in the Mediterranean. The ARMed project investigated the epidemiology of antimicrobial resistance, as well as its contributory factors, in a number of countries in the southern and eastern Mediterranean region through the collection of comparable and validated data. The methods used to collect data were: susceptibility test from invasive isolates, structured questionnaires and structured interviews.

The Global Antimicrobial Resistance Surveillance System (GLASS) is being launched by WHO and implemented in 2015 to support a standardized approach to the collection, analysis and sharing of data on AMR at a global level, in order to inform decision-making, drive local, national and regional action, and provide the evidence base for action and advocacy. GLASS aims to combine clinical, laboratory and epidemiological data on pathogens that pose the greatest threats to health globally. The GLASS manual details the proposed approach for the early implementation of the surveillance system, that focus on antibiotic-resistant bacteria, and outlines the flexible and incremental development of the system that incorporates over time lessons learned from the early implementation phase.^1^ Antibacterial class and antibacterial agents evaluated by GLASS are shown in the table 1, available in the related Manual for Early Implementation.^2^

Another source considered is EARS-Net, a European network of national surveillance systems that maintains a comprehensive monitoring and information system with European reference data on antimicrobial resistance for public health purposes. EARS-Net, coordinated from 2010 by the European Centre for Disease Prevention and Control, is the largest publicly funded surveillance system for antimicrobial resistance in the European region. The results contribute to greater public awareness and scientific understanding of antimicrobial resistance and its importance in public health. Antimicrobial resistance in Europe is monitored by a network of national surveillance systems in the European countries.

The national networks systematically collect data from clinical laboratories in their own countries and upload the data to a central database maintained at European Centre for Disease Prevention and Control (The European Surveillance System - TESSy). After uploading, each country approves its own data and the results are made available from the ECDC website.^3^ In order to maintain and facilitate the data reporting, ECDC ensures: validation of data from antimicrobial susceptibility tests for seven important bacterial pathogens isolated from patients with invasive infections; analysis of trends in the occurrence of antimicrobial resistance over time and in different countries and regions; EQA (External Quality Assessment) and protocols on testing methods to improve the consistency and quality of the data.

Instead, ESAC-Net (formerly ESAC) is a Europe-wide network of national surveillance systems, providing European reference data on antimicrobial consumption. ESAC-Net collects and analyses data on antimicrobial consumption from EU and EEA/EFTA countries, both in the community and in the hospital sector.

The coordination of ESAC-Net was transferred to the European Centre for Disease Prevention and Control in July 2011.

The collected data are used to provide timely information and feedback to EU and EEA/EFTA countries on indicators of antimicrobial consumption. These indicators provide a basis for monitoring the progress of EU and EEA/EFTA countries towards the prudent use of antimicrobials.^4^

## Discussion

In July 2006 the working group leading the “ARMed project” has outlined the preliminary results of their work, referring in particular to antibiotic resistance in the southeastern Mediterranean. The results were as follows: sporadic reports from centers in the south and east of the Mediterranean have suggested that the prevalence of antibiotic resistance in this region appears to be considerable, yet pan-regional studies using comparable methodology have been lacking in the past. Susceptibility test results from invasive isolates of *Staphylococcus aureus*, *Streptococcus pneumoniae*, *Escherichia coli*, *Enterococcus faecium* and *faecalis* routinely recovered from clinical samples of blood and cerebrospinal fluid within participating laboratories situated in Algeria, Cyprus, Egypt, Jordan, Lebanon, Malta, Morocco, Tunisia and Turkey. The samples, collected as part of the ARMed project Preliminary data in the first two years of the project, showed the prevalence of penicillin non-susceptibility in *Streptococcus pneumoniae* ranging from 0% (Malta) to 36% (Algeria) [median: 29%], whilst methicillin resistance in *Staphylococcus aureus* varied from 10% in Lebanon to 65% in Jordan [median: 43%]. Country specific significant resistance in *Escherichia coli* was also investigated. 72% of isolates reported from Egyptian hospitals was resistant to third generation cephalosporins and 40% was non-susceptible to fluoroquinolones in Turkey. Vancomycin non-susceptibility was reported only in 0.9% of *Enterococcus faecalis* isolates from Turkey and in 3.8% of *Enterococcus faecium* isolates from Cyprus. The preliminary results from the ARMed project appear to support previous sporadic reports suggesting high antibiotic resistance in the Mediterranean region. They suggest that this is particularly the case in the eastern Mediterranean region where resistance in *Staphylococcus aureus* and *Escherichia. coli* seems to be higher than that reported in the other countries of the Mediterranean.^5^

A publication of March 2007 reports the results of a survey of infection control infrastructure in selected southern and eastern Mediterranean hospitals. A structured questionnaire concerning hospital infection control (IC) organization and initiatives was sent to 45 hospitals in Algeria, Cyprus, Egypt, Jordan, Lebanon, Libya, Malta, Morocco, Tunisia and Turkey. Hospitals bordering the eastern Mediterranean appeared to have more established IC infrastructures than southern Mediterranean hospitals. However, there were no significant differences among hospitals in the two regions in surveillance activities, the presence of an antibiotic policy or feedback of resistance data to prescribers, all of which were at a low level. Only a minority of hospitals had published antimicrobial treatment guidelines or gave feedback on antimicrobial resistance data to prescribers.^6^

A further relevant work by the same authors is that of December 2007 concerning the prevalence of methicillin-resistant *Staphylococcus aureus* (MRSA) in invasive isolates from southern and eastern Mediterranean countries. In this work, it appears that most of the countries in the Mediterranean region are experiencing a surge in MRSA infections. This requires a greater focus to identify relevant drivers of resistance and implement effective practices consistent especially with improved infection control and antibiotic consumption practices.^7^

In August 2008, the same group addressed the issue of antimicrobial resistance in invasive strains of *Escherichia coli* from southern and eastern Mediterranean laboratories. From January 2003 to December 2005, 5091 susceptibility test results from invasive isolates of *Escherichia coli*, collected from blood cultures and cerebrospinal fluid routinely processed within 58 participating laboratories, were investigated. These laboratories in turn serviced 64 hospitals in Algeria, Cyprus, Egypt, Jordan, Lebanon, Malta, Morocco, Tunisia and Turkey. The median proportion of resistance to third-generation cephalosporins for the duration of the project was 18.9% (interquartile range (IQR): 12.5–30.8%), and for fluoroquinolones 21.0% (IQR: 7.7–32.6%). A substantial proportion of strains reported by laboratories in countries east of the Mediterranean exhibited evidence of multiresistance, the highest percentage being from Egypt (31%).^8^

In October 2008, the same working group described the characteristics of antibiotic consumption in southern and eastern Mediterranean hospitals. Emphasis on wide-spectrum agents could explain one possible factor behind the documented high prevalence of resistance in important pathogens within these same hospitals and suggests the need for improved antibiotic stewardship and prescribing programs, which may well apply to the whole region.^9^

In November 2008, the same group has drawn up a work concerning the Infection control and antibiotic stewardship practices reported by southeastern Mediterranean hospitals. The prevalence of multiple resistant organisms (MROs) reported from south-eastern Mediterranean hospitals highlights the need to identify possible contributory factors to help design control interventions. This aspect was investigated through a structured questionnaire, which examined infection control and antibiotic stewardship practices in hospitals participating or collaborating with the Antibiotic Resistance Surveillance & Control in the Mediterranean Region (ARMed) project.

A total of 45 hospitals (78.9% of invited institutions) responded to the questionnaire; 60% indicated that they faced periods of overcrowding when available bed complement was insufficient to cope with hospital admissions and 62% reported difficulties in isolating patients with MROs due to lack of available beds. Most hospitals relied mainly on washing to achieve hand hygiene, whether by non-medicated or disinfectant soaps. Dependence on solid bars of soap (28.9%) and cloth towels (37.8%) were among the problems identified as well as inconvenient distances of sinks from patient beds (66.6%). Alcohol hand rub was the effective hand hygiene product in only 7% of hospitals. Programs for better antibiotic use were mostly limited in scope; 33.3% reported having antibiotic prescribing guidelines, and 53.3% of hospitals fed back resistance rates to prescribers. Auditing of antibiotic consumption, whether institution- or unit-based, was carried out in 37.8% of responding hospitals. Multi-faceted approaches to improving the isolation of patients with ORP, increasing the emphasis on hand hygiene, encouraging greater use of alcohol hand rubs and the introduction of effective antibiotic management programs should be more widespread in southeastern Mediterranean hospitals.^10^ In January 2009, this working group has studied the correlation between methicillin-resistant *Staphylococcus aureus* prevalence and infection control initiatives within southern and eastern Mediterranean hospitals. The Mediterranean region has been identified as an area of hyperendemicity for multi-resistant hospital pathogens. To better understand the potential drivers behind this situation, the group attempted to correlate the previously published methicillin-resistant Staphylococcus aureus (MRSA) data from 27 hospitals, participant in the Antibiotic Resistance Surveillance & Control in the Mediterranean Region (ARMed) project, with the responses received from the same institutions to questionnaires which dealt with various aspects of infection control and antibiotic stewardship. No difference in the scores replies to structured questions regarding infection control set-up, hand hygiene facilities and antibiotic stewardship practices could be ascertained between hospitals with a high or low prevalence of infections. However, the group did identify differences concerning bed occupancy and isolation facilities. Hospitals reporting frequent episodes of overcrowding, particularly involving several departments, and which found persistent difficulties in sourcing isolation beds, had significantly higher MRSA proportions. This suggests that infrastructural deficits related to insufficient bed availability and compounded by inadequate isolation facilities could potentiate MRSA hyperendemicity in south-eastern Mediterranean hospitals.^11^

In 2009, the same group described the role of the Self-medication with antibiotics in the ambulatory care setting within the Euro-Mediterranean region. Anecdotal data from the southern and eastern Mediterranean region suggests that self-medication with antibiotics is commonly practiced in many countries. Thus, non-prescribed antibiotic use is high within ambulatory care in the south and east Mediterranean countries, being almost twice that reported in a similar European study. Corrective efforts are clearly required in the region to ensure proper use of antimicrobials so as to reduce pressure for antimicrobial resistance. In order to provide adequate information on the situation, the group undertook short structured interviews in out-patients clinics or primary health centers in Cyprus, Egypt, Jordan, Lebanon, Libya, Tunisia and Turkey. A total of 2109 interviews were undertaken of which 1705 completed the full questionnaire. Self-medication was reported by 19.1% (<0.1% in Cyprus to 37% in Lebanon) of respondents. Intended self-medication ranged from 1.3% (95% CI 0%, 3%) in Cyprus to 70.7% (95% CI 64%, 77%) in Jordan. Upper respiratory tract symptoms were the most frequent reasons for which respondents indicated they would self-medicate. 48.4% of the whole group replied that they kept antibiotics at home, being highest in Lebanon (60%, 95% CI 51%, 69%). The group found a significant association between antibiotic hoarders and intended users of antibiotics for self-medication.^12^

In March 2009 the same group described the prevalence of penicillin and erythromycin resistance among invasive *Streptococcus pneumoniae* isolates reported by laboratories in the southern and eastern Mediterranean region. Information about the epidemiology of resistance in *Streptococcus pneumoniae* within these countries of the Mediterranean region is incomplete, as reports have been sporadic and difficult to compare. ARMed data on the antimicrobial resistance epidemiology of *Streptococcus pneumoniae* in the southern and eastern Mediterranean region provided evidence of high rates of resistance, especially to penicillin. This evidence calls for a greater focus on the identification of relevant drivers of resistance and on the implementation of effective practices to address this problem. Over a 36-month period, from 2003 to 2005, the ARMed project collected 1298 susceptibility test results of *Streptococcus pneumoniae* invasive isolates from blood and spinal fluid cultures routinely processed within 59 participating laboratories situated in Algeria, Cyprus, Egypt, Jordan, Lebanon, Malta, Morocco, Tunisia and Turkey. Overall, 26% (335) of isolates were reported as non-susceptible to penicillin, with the highest percentages being reported from Algeria (44%) and Lebanon (40%). During the same period, the highest proportions of pneumococci that were not susceptible to erythromycin were reported from Malta (46%) and Tunisia (39%). Dual non-susceptibility proportions more than 5% were found in laboratories of Algeria, Tunisia, Lebanon, Jordan and Turkey.^13^

Some consideration and conclusion have been drawn from the ARMed project throughout the years. The consensus conference held in Malta in November 2006 provided a forum for expert delegates to agree on some priority strategic recommendations that would be relevant to resistance containment efforts in the region. There was general agreement on the need for surveillance and audit to underpin any intervention to tackle antimicrobial resistance, both to monitor changing epidemiological trends in critical pathogens as well as to identify antibiotic consumption practices and effectiveness of prevention and control of health care-associated infections. In October 2009, the working group reported the Consensus Conference of the Antibiotic Resistance Surveillance and Control Group in the Mediterranean region. Antimicrobial resistance was considered a global threat to effective health care delivery. However the case in the Mediterranean area the data from recent studies suggested a situation of overwhelming danger. A better knowledge base, as well as a collaborative effort, was therefore required to address this increasing challenge to an effective patient care. The importance to convey these data to key users was also stressed in all workshops, as was better education of patients and training of health care workers. The recommendations also made it clear that ownership of the problem needs to be improved throughout the involvement of single regions and nations and that specific resources, both financial as well as human, must be allocated by the respective policy makers to counteract it.^14^

In April 2010 the working group has drawn up a work concerning Antibiotic consumption as a driver for resistance in *Staphylococcus aureus* and *Escherichia coli* within a developing region. The results suggest an association between resistance and antibiotic use, especially for carbapenems and a third-generation cephalosporin. These data support the urgent implementation of antibiotic stewardship initiatives in hospitals in developing countries that focus on a more judicious use of broad-spectrum formulations.^15^

In addition, in 2012 the European Centre for Disease Prevention and Control (ECDC) launched the ‘European survey of carbapenemase-producing Enterobacteriaceae (EuSCAPE)’ project to gain insights into the occurrence and epidemiology of carbapenemase-producing *Enterobacteriaceae* (CPE), to increase the awareness of the spread of CPE, and to build and enhance the laboratory capacity for diagnosis and surveillance of CPE in Europe. Data collected through a post-EuSCAPE feedback questionnaire in May 2015 documented improvement compared with 2013 in capacity and ability to detect CPE and identify the different carbapenemases genes in the 38 participating countries, thus contributing to their awareness of and knowledge about the spread of CPE. Over the last two years, the epidemiological situation of CPE worsened, in particular with the rapid spread of carbapenem-hydrolysing oxacillinase-48 (OXA-48)- and New Delhi Metallo-beta-lactamase (NDM)-producing Enterobacteriaceae. In 2015, 13/38 countries reported inter-regional spread of or an endemic situation for CPE, compared with 6/38 in 2013. Only three countries replied that they had not identified one single case of CPE. The ongoing spread of CPE represents an increasing threat to patient safety in European hospitals, and a majority of countries reacted by establishing national CPE surveillances systems and issuing guidance on control measures for health professionals. However, 14 countries still lacked specific national guidelines for prevention and control of CPE in mid-2015.^16^

ECDC (European Centre for Disease Prevention and Control) in November 2015 has published a report on the supervision on antibiotic resistance in the year 2014. For different combinations bacterium-drug reported an increase of resistance as you move from North to South and from West to East of Europe; This is due to the differences between the different zones, the appropriateness of the use of antibiotics and health care practices, as well as in the effectiveness of infection control.

The phenomenon of resistance presented by some gram-negative, specifically focusing on resistance of *Klebsiella pneumoniae* and *Escherichia coli* to third-generation cephalosporins, often combined in opposition to fluoroquinolones and aminoglycosides, appears very dangerous. It can lead to an increased use of carbapenems which can in turn promote resistance to this class of antibiotics: this has been highlighted much more to Klebsiella pneumoniae. In fact, from the data available, is particularly relevant the incidence of resistance to carbapenems by the strains of *Enterobacteriaceae* (*Klebsiella* included). Manufacturer of carbapenemase.

Carbapenem resistance is also common (together with opposition to groups of antibiotics) on *Pseudomonas aeruginosa* and *Acinetobacter spp*. In these cases, treatment options include the use of drug combinations and the use of old antibiotics like polymyxin and colistin (to which are still common strengths, particularly the first).

**Figure 1:**
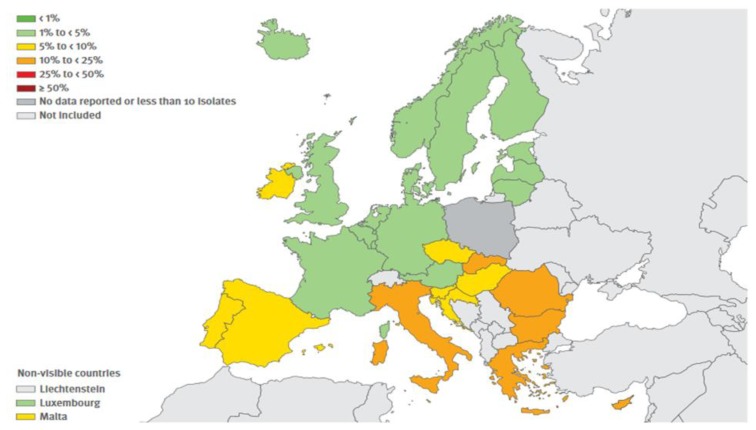
(Antimicrobial resistance surveillnace in Europe, Surveillance report, ECDC, 2014). *Escherchia coli.* Percentage (%) of invasive isolate with combined resistance to third generation cephalosporin, fluorochinolones and aminoglycosides, by country, EU/EEA countries, 2014

**Figure 2:**
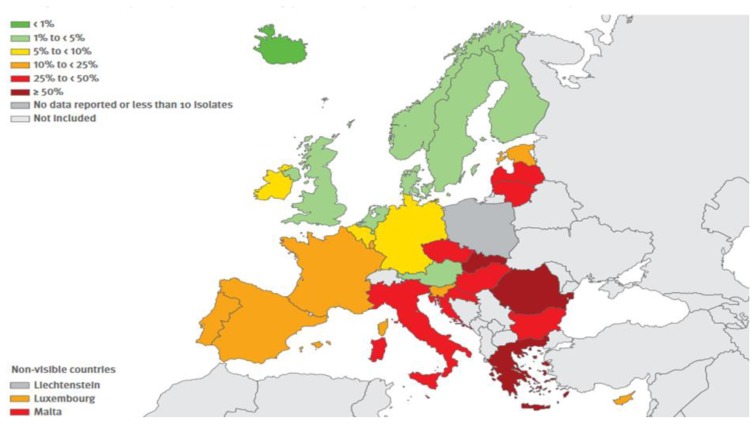
(Antimicrobial resistance surveillnace in Europe, Surveillance report, ECDC, 2014). *Klebsiella pneumoniae* Percentage (%) of invasive isolates with combined resistance to fluoroquinolones, third generation cephalosporins and aminoglycosides, by countries, 2014.

**Figure 3:**
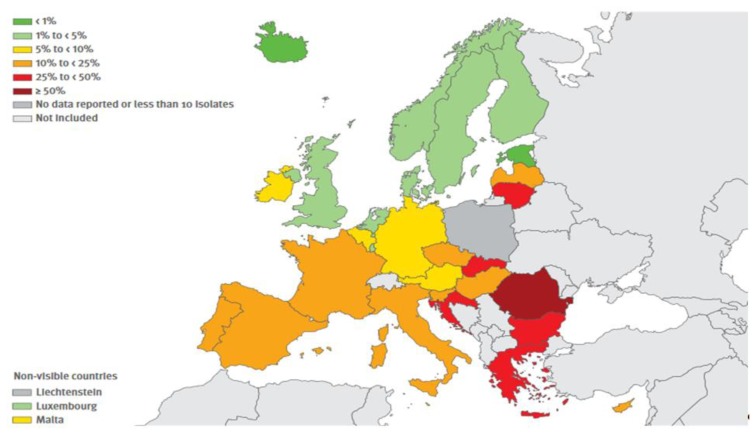
(Antimicrobial resistance surveillnace in Europe, Surveillance report, ECDC, 2014). *Pseudomonas aeruginosa*. Percentage (%) of invasive isolates with combined resistance (resistance to three or more antimicrobial groups among piperacillin + tazobactan, ceftazidime, fluoroquinolones, amynoglycosides and carbapenems), by country, EU/EEA countries, 2014.

**Figure 4:**
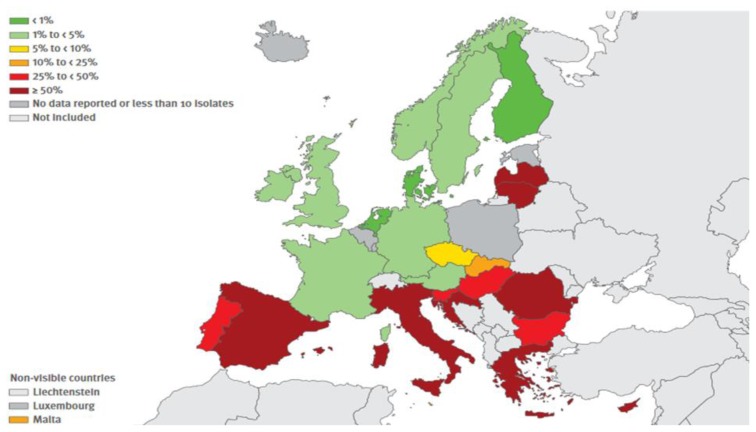
(Antimicrobial resistance surveillnace in Europe, Surveillance report, ECDC, 2014). *Acinetobacter spp*. Percentage (%) of invasive isolates with combined resistance to fluoroquinolones, aminoglycosides and carbapenens, by country, EU/EEA countries, 2014.

The resistance exerted by gram-positive shows a more mixed picture. The resistance exerted by MRSA (Methicillin-resistant *Staphylococcus aureus*) continues to be relevant, albeit showing some decline in recent years whereas that carried on by *Streptococcus pneumoniae* is stable and is mainly relevant to macrolides. Finally, it is significant the increase of resistance exercised by *Enterococcus faecium* to Vancomycin. The situation in Europe concerning combined resistances exerted by certain bacteria, are shown in Figures 1, 2, 3 and 4.^17^ It is clear that, from this point of view, the situation in Northern Europe is better than that of the countries bordering the Mediterranean, with particular reference to what concerns *Escherichia coli*, *Klebsiella pneumoniae*, *Pseudomonas aeruginosa* and *Acinetobacter spp*.

## Conclusions

In May 2015, the Sixty-eighth World Health Assembly (the decision-making body of WHO) adopted the global action plan on antimicrobial resistance. One of the five strategic objectives of the action plan is to strengthen the evidence base through enhanced global surveillance and research.^1^

Antimicrobial resistance (AMR) surveillance is the cornerstone for assessing the burden of AMR and for providing the necessary information for action in support of local, national and global strategies. The Global Antimicrobial Resistance Surveillance System (GLASS) is being launched to support a standardized approach to the collection, analysis and sharing of data on AMR at a global level, to inform decision-making, drive local, national and regional action, and provide the evidence base for action and advocacy.

Countries can benefit from participation in GLASS through enhanced capacity building, access to training and implementation tools, and support in collecting AMR data at local and national levels. Country participation in GLASS must be with the agreement of the national government.

Early implementation of GLASS is based on this type of surveillance. A benefit of the monitoring approach proposed in GLASS is that epidemiological and clinical data are combined with microbiological data. This system allows stratification of populations to ascertain e.g. the most frequent type of resistant infection, the age structure of infections, whether most occur in the community or hospital and the geographical distribution. This method has also drawbacks. Although infections due to bacteria resistant to antimicrobial agents do not usually present differently from those due to the same but susceptible bacteria, in the settings where samples are not routinely sent for microbiological investigation, those examined are more likely to be taken from severely ill patients who have failed first- and perhaps second-line treatment. This type of surveillance is therefore more likely to find resistant strains. Despite these caveats, data from surveillance based on routinely collected clinical samples can be used for several purposes if core data are collected both on patients and the population from which they derive and if duplicated results for the same patient are removed:

- A combination of epidemiological and laboratory data allows stratification of populations for ascertaining the type of infection, whether most AMR infections are occurring, e.g. in young or elderly people, in the community or hospital, and reporting the geographical distribution of infections caused by resistant organisms;
- The extent of AMR infections can be assessed from epidemiological indicators such as incidence and prevalence. Epidemiological and laboratory data must represent a defined population to be pertinent. For infections of hospital origin, the most common denominator is the number of hospital admissions or patient-days in the hospital. The population denominator for assessing infections occurring in the community may be difficult to determine, as it requires counting the number of infections in a population in a specified catchment area. However, it is crucial for understanding variations in sampling frequency and impact of resistant infections;
- Routine data from antibiograms can be analyzed for new trends by comparing them with data from the previous year, to determine any significant change in important resistant bacteria, such as carbapenem-resistant *Enterobacteriaceae*. Information on any increase is critical for designing local measures to prevent transmission of resistant bacteria; a decrease may reflect an impact of interventions. As one of the objectives of early implementation is to build common understanding by testing the feasibility of collecting and sharing harmonized data, the limitations of surveillance based on routinely collected clinical samples is not considered to be a major problem at this stage.^2^

In reference to the prospects, the World Alliance Against Antibiotic Resistance (WAAAR) is a nonprofit organization open to professionals and consumers worldwide. It constitutes a group of 700 individuals from 55 different countries representing all the key stakeholders (physicians, veterinarians, microbiologists, surgeons, pharmacists, nurses, evolutionary biologists, ecologists, environmentalists, and patient advocacy groups). The Alliance receives support from more than 140 scientific societies or professional groups throughout the world.^18^

The cost of antimicrobial resistance (AMR) entails 300 million premature deaths (every year: 390.000 deaths in Europe, 4.150.000 deaths in Africa, 4.730.000 in Asia) and up to $100 trillion (£64 trillion) lost to the global economy by 2050.^19^ Detecting, preventing, and controlling antibiotic resistance requires strategic, coordinated, and sustained efforts. It also depends on the engagement of governments, academia, industry, healthcare providers, the general public, and the agricultural community, as well as international partners. Committing to combating antibiotic-resistant microbes supports patient care, economic growth, agriculture, and economic and national security.^20^

## Figures and Tables

**Table 1:** Pathogen–antimicrobial combinations on which GLASS will gather data (Adapted by the Manual for Early Implementation, GLASS).

Pathogen	Antibacterial class	Antibacterial agents that may be used for AST^a^,^b^

*Escherichia coli*	Sulfonamides and trimethoprim	Co-trimoxazole
	Fluoroquinolones	Ciprofloxacin or levofloxacin
	Third-generation cephalosporins	Ceftriaxone or cefotaxime and ceftazidime
	Fourth-generation cephalosporins	Cefepime
	Carbapenems^c^	Imipenem, meropenem, ertapenem or doripenem
	Polymyxins	Colistin
	Penicillins	Ampicillin

*Klebsiella pneumoniae*	Sulfonamides and trimethoprim	Co-trimoxazole
	Fluoroquinolones	Ciprofloxacin or levofloxacin
	Third-generation cephalosporins	Ceftriaxone or cefotaxime and ceftazidime
	Fourth-generation cephalosporins	Cefepime
	Carbapenems^c^	Imipenem, meropenem, ertapenem or doripenem
	Polymyxins	Colistin

*Acinetobacter baumannii*	Tetracyclines	Tigecycline or minocycline
	Aminoglycosides	Gentamicin and amikacin
	Carbapenems^c^	Imipenem, meropenem, ertapenem or doripenem
	Polymyxins	Colistin

*Staphylococcus aureus*	Penicillinase-stable beta-lactams	Cefoxitin^d^

*Streptococcus pneumoniae*	Penicillins	Oxacillin^e^
		Penicillin G
	Sulfonamides and trimethoprim	Co-trimoxazole
	Third-generation cephalosporins	Ceftriaxone or cefotaxime

*Salmonella* spp.	Fluoroquinolones	Ciprofloxacin or levofloxacin
	Third-generation cephalosporins	Ceftriaxone or cefotaxime and ceftazidime
	Carbapenems^c^	Imipenem, meropenem, ertapenem or doripenem

*Shigella* spp.	Fluoroquinolones	Ciprofloxacin or levofloxacin
	Third-generation cephalosporins	Ceftriaxone or cefotaxime and ceftazidime
	Macrolides	Azithromycin

*Neisseria gonorrhoeae*	Third-generation cephalosporins	Cefixime
		Ceftriaxone
	Macrolides	Azithromycin
	Aminocyclitols	Spectinomycin
	Fluoroquinolones	Ciprofloxacin
	Aminoglycosides	Gentamicin

a The listed substances are priorities for surveillance of resistance in each pathogen, although they may not be first-line options for treatment. One or more of the drugs listed may be tested.

b One or more of the drugs listed may be tested in countries. S, I, R and nominator and denominator data for each shall be reported separately.

c Imipenem or meropenem is preferred to represent the group when available.

d Cefoxitin is a surrogate for testing susceptibility to oxacillin (methicillin, nafcillin); the AST report to clinicians should state susceptibility or resistance to oxacillin.

e Oxacillin is a surrogate for testing reduced susceptibility or resistance to penicillin; the AST report to clinicians should state reduced susceptibility or resistance to penicillin.
